# Josué de Castro, autor de livro didático de geografia

**DOI:** 10.1590/S0104-59702025000100064

**Published:** 2026-02-09

**Authors:** Breno Viotto Pedrosa

**Affiliations:** i Professor, Universidade Federal do Rio Grande do Sul; membro pesquisador, Instituto Histórico e Geográfico do Rio Grande do Sul. Porto Alegre – RS – Brasil. brenoviotto@hotmail.com

**Keywords:** Josué Apolônio de Castro (1908-1973, Livro didático, Geografia humana, História da geografia, Circulação do conhecimento, Josué Apolônio de Castro (1908-1973, Textbook, Human geography, History of geography, Circulation of knowledge

## Abstract

Analisa-se *Geografia humana: estudo da paisagem cultural do mundo*, livro didático de Josué de Castro, a partir da teoria de Pierre Bourdieu. Busca-se elucidar a atuação profissional e acadêmica do intelectual brasileiro durante a década de 1930, explorando as estratégias didáticas, os interlocutores internacionais e o processo de editoração do livro. Demonstra-se que o manual de Castro é uma obra original por problematizar raça e clima como fatores explicativos da geografia humana e por ser sensível à cultura popular. Ressalta-se sua erudição no campo da geografia com a mobilização de conceitos que são elaborados e problematizados, como raça, aclimatação e aculturação em diálogo com a antropologia. A análise indica ideias que serão desenvolvidas em sua obra *Geografia da fome*.

## Abstract (en)

This text examines Josué de Castro’s Geografia humana: estudo da paisagem cultural do mundo, based on the theory of Pierre Bourdieu. We investigate Castro’s professional and academic activity during the 1930s, focusing on his educational strategies, international connections, and the text’s editing process. Castro’s work is shown to be unique for its critique of race and climate as determinants for human geography and its sensitivity to popular culture. Our analysis highlights the text’s contribution to geography, with the development and questioning of concepts like race, acclimatization and acculturation, in dialog with anthropology. We also note ideas that appear later in Castro’s book Geografia da fome.

Parece estranho, mas é pura verdade. Vim saturado do espírito europeu, um espírito que cai aos poucos. O sul-americano que chega naquelas plagas, principalmente o brasileiro, logo nota que alguma coisa está apodrecendo no Velho Mundo. A inteligência está cansada. A habilidade e a astúcia vão ocupando os lugares ontem pertencentes ao espírito criador e ao talento lúcido. Não sei, não. Mas não queria morar lá, nem como rei de qualquer monarquia transitória...

Josué de Castro, em 1939, ao retornar da Itália.

Josué Apolônio de Castro (1908-1973) é uma fonte inesgotável de inspiração e estudos. O objetivo deste artigo é explorar uma faceta pouco analisada de Castro, um livro didático intitulado *Geografia humana: estudo da paisagem cultural do mundo*, publicado, em 1939, pela editora do Globo de Porto Alegre. Pouca atenção foi desprendida na avaliação e contextualização desse manual, destinado ao, na época, terceiro ano do curso secundário. Muito já foi escrito sobre Josué de Castro, cientista da fome no Brasil e no mundo, ou ainda sobre sua atuação política, seu papel em órgãos internacionais; contudo, a revisão desse livro didático revela uma nova faceta do personagem. Castro nunca lecionou para discentes do ensino básico ou do secundário, ou seja, fora da universidade, e ele redigiu o livro didático quando atuava como professor de antropologia física na então Universidade do Distrito Federal.

Adotaremos, assim, aquilo que Berdoulay (2003) chamou de abordagem contextual, ou seja, uma averiguação das circunstâncias de publicação do livro, combinado com uma perspectiva que se debruça sobre seu conteúdo e sua arquitetura. Lira (2021) ressaltou a característica da escola francesa de ter um método implícito, e Castro, influenciado por esse grupo, pode ter seguido a tendência. Veremos que na *Geografia humana*, em nome da didática, o autor elucida sinteticamente alguns pontos-chave de sua geografia. Este estudo se justifica, pois o manual é a publicação que antecedeu o lançamento da *magnum opus* de Castro, *Geografia da fome*, de 1946, impressa por *O Cruzeiro* do Rio de Janeiro. Embasa ainda este artigo a teoria dos campos de Pierre Bourdieu (2001, 2003, 2011), que nos ajuda a compreender a posição de Castro no campo da geografia brasileira na década de 1930, momento importante para a institucionalização da disciplina. O conceito de capital social é fundamental para compreender Castro nessa década, pois, apesar de jovem, Castro era uma espécie de intelectual público, bem relacionado e com presença cativa nas colunas dos jornais, tanto no noticiário social como engajando-se no debate sobre fome, alimentação e salário-mínimo.

Os livros didáticos de geografia e história, no Brasil, no início do século XX, frequentemente eram publicados por membros de sociedades geográficas que lecionavam em escolas normais, colégios militares, ginásios, no Colégio Pedro II, sendo que alguns desses intelectuais, por vezes, também publicavam trabalhos acadêmicos e, em alguns casos, converteram-se em professores universitários. O Colégio Pedro II era um centro prestigiado em que vários docentes se tornaram referência nacional e passaram para o ensino superior; ademais, seus currículos foram aplicados para todo o Brasil. Josué de Castro participou esparsamente das sociedades geográficas, e Antunes (2023, p.76) salienta que, em 1939, em reunião da Associação dos Geógrafos Brasileiros (em São Paulo), ele proferiu palestra sobre “Os mocambos do Nordeste”. Ferretti (2022) destaca Pierre Monbeig como a figura por trás do convite e identifica um conjunto de cartas trocadas entre ambos. Nesse sentido, parece que Castro fez o caminho inverso do habitual para época, pois já era docente do ensino superior e escreveu um manual para os alunos do curso secundário. Resta compreender melhor as circunstâncias de seu manual, pois Castro tinha uma formação de médico e uma trajetória interdisciplinar, mas aspira reforçar sua identificação com a geografia, mesmo ocupando cargo na antropologia.

## Recife, Rio de Janeiro e Porto Alegre

Seria supérfluo rememorar a biografia de Castro, trabalho já feito por uma série de pesquisadores (Amorim, 2022; Silva, 2012; Mendonça, 2021; Carvalho, 2008). Cabe, entretanto, voltar pontualmente à sua juventude: Bourdieu (2011) ressaltou a importância da formação informal, ou seja, mais importante do que o acesso a uma educação escolar, o que se aprende em casa é relevante, o que permite ter uma compreensão das regras formais e informais da vida social, além dos contatos, o que facilita a conversão do capital cultural em social. Castro teve origens humildes, e, no contexto do Nordeste brasileiro da década de 1920, o estudo de medicina apresentava uma perspectiva de ascensão social. Pouco relevo foi dado ao fato de seu pai ser um sertanejo empobrecido, mas a “Mamãe era de família de engenho, aristocrata, Carneiro da Cunha” (Mendonça, 2021) e professora primária. Bourdieu insistiu no argumento de que uma das estratégias da burguesia para enfrentar a decadência do capital econômico é sua conversão em capital cultural.

Os relatos memorialistas dão conta de descrever que Castro passou por dificuldades e, em entrevista (Entrevista..., 27 abr. 1939, p.8), ele conta que morava em um velho casarão no Recife, ao lado dos mocambos, onde via a pobreza, os que sobreviviam do caranguejo, local em que ocorriam as festas populares. De acordo ainda com o relato, os pais se separaram quando ele era jovem e não tinham uma boa relação; entretanto, a cultura da mãe e sua origem familiar podem ter sido um trunfo para sua ascensão no sentido de ter-lhe ofertado uma cultura geral, domínio da forma de se portar em diferentes situações sociais e, sobretudo, contatos, ou seja, um capital social. Sua situação de pobreza não impediu que ele frequentasse os colégios tradicionais de Recife, destacando sua propensão a uma articulação internacional, pois, logo após se formar no curso de medicina, na cidade do Rio de Janeiro, em 1929, Castro viaja para o México representando entidade estudantil e faz estágio em medicina na Universidade de Columbia (Machado, Alves, Azevedo, 2010).

Cabe destacar que, no início de sua formação, na Bahia, Castro convive com Arthur Ramos, um colaborador profícuo que, apesar de médico, se especializa na antropologia (Ferretti, 2022). Na juventude estudantil na Bahia e no Rio de Janeiro, Castro estabelece uma rede de amigos artistas e literatos, escreve sobre cinema e contos. Melo (1991) destaca a proximidade com os artistas que permeia toda sua trajetória, travando relações com Mario de Andrade, Rachel de Queiroz, Cândido Portinari, Jorge Amado, entre outros.

Em 1932, Castro defende sua livre-docência na Faculdade de Medicina do Recife, cujo tema é o problema da alimentação, trabalho que é criticado por Gilberto Freyre e suscita uma resposta pública (Melo, 1991, p.133). Nesse contexto, Castro foi um dos responsáveis pela fundação da Faculdade de Filosofia e Ciências Sociais em Recife, onde atuou como professor de geografia humana de 1933 a 1935. Após o fechamento dessa instituição, segundo Castro, devido a alguns “reacionários” que obstaculizaram seu funcionamento, ele se muda para o Rio de Janeiro, buscando contornar dificuldades financeiras (Amorim, 2022). Segundo Barros (2012) e Melo (2012), Castro estava engajado no grupo político de José Maria Belo, aspirante ao governo de Pernambuco, que lhe havia prometido uma vaga na Secretaria da Educação; contudo, o golpe de 1930 anula os planos. Paralelamente, Barros (2012) defende que a ascensão do médico pernambucano Pedro Ernesto à prefeitura do Distrito Federal foi um chamariz para Castro e outros intelectuais pernambucanos, como Gilberto Freyre, que aspiravam participar de um governo de mudança social. Pedro Ernesto, no entanto, cai em desgraça após a intentona comunista de 1935 (Barros, 2012, p.245).

Em meados da década de 1930, na cidade do Rio de Janeiro, então capital do país, Castro não hesita em contatar Anísio Teixeira, que era Secretário de Educação do Rio de Janeiro. A demanda de Castro era por um posto na universidade que lhe havia sido prometido pelo positivista pernambucano, docente de química e funcionário do Ministério da Agricultura, Paulo Estevão Berredo Carneiro (1901-1982) – novamente relações que denotam seu capital social. Segundo Amorim (2022), é pela mão de Roquette Pinto que Castro se torna professor de antropologia física na então Universidade do Distrito Federal, sendo que Pinto havia sido seu professor na Faculdade de Medicina do Rio de Janeiro. Cabe destacar que Castro, antes de lecionar, já era uma figura notável, que desfrutava de um significativo capital cultural: durante a juventude, no Recife, ele havia publicado comentários literários e contos nos jornais, além de ter lançado, em 1933, o livro *O problema da alimentação no Brasil* (Castro, 1933), que recebeu comentário não assinado no jornal carioca *A Noite* (Alimentação..., 7 ago. 1934), e o livro *Alimentação e raça* (Castro, 1935), que recebeu críticas positivas no jornal *Diário Carioca:* “Alimentação e raça” (Carneiro, 15 dez. 1935); “Um livro patriótico” (Mendes, 15 dez. 1935); “Alimentação e raça” (Pinto, 12 jan. 1936); “Alimentação e raça” (Campello, 2 fev. 1936). Tais comentários consagram Castro como especialista no tema, envolvendo profissionais do campo da medicina e da antropologia como Roquette Pinto, que prefaciou *Alimentação e raça,* integrante da coleção biblioteca de divulgação científica, dirigida por seu amigo Arthur Ramos. Como demonstrado por Vasconcelos (2001), Leme (2021) e Bizzo (2009), Castro, nesse período, é um sintetizador do debate sobre a fome. Descartando a explicação racial de cunho eugênico ou pautada no determinismo climático, o autor constrói uma visão socioeconômica da fome, cuja renda das famílias e o elemento ecológico são os fatores causais.

Castro parece ter feito uma mistura catalisadora para o crescimento do seu capital cultural: era alguém engajado no campo literário, na medicina, na antropologia e na geografia, com relações relevantes no Recife e no Rio de Janeiro; era um jovem cientificamente competente que precocemente se preocupou com a questão da nutrição em um país onde se comia mal. Munido desse capital social, Castro começou a ser visto como um reconhecido especialista da nutrição, temática que acabou sendo relacionada à questão nacional e ao desenvolvimentismo (Bizzo, Lima, 2010), tendo nosso autor assumido uma postura cética quanto às teorias raciais, defendendo que o problema do trabalhador era a alimentação, e não a “raça” – um antídoto ao pensamento científico que havia se consolidado no fim do século XIX (Schwarcz, 1993). O resultado disso é um número bastante expressivo de artigos que o mencionam ou são de sua autoria nos jornais cariocas. Para demonstrar sua relevância no debate público, escolhemos os jornais *ANoite* e o *Diário Carioca* e selecionamos qualquer tipo de notícia que mencionasse o nome “Josué de Castro”, excluindo os anúncios de seu consultório, uma vez que Castro clinicava. Em algum momento, durante a década de 1930, a filha de Getúlio Vargas se torna sua paciente. O resultado foi o seguinte:

**Figura 1:**
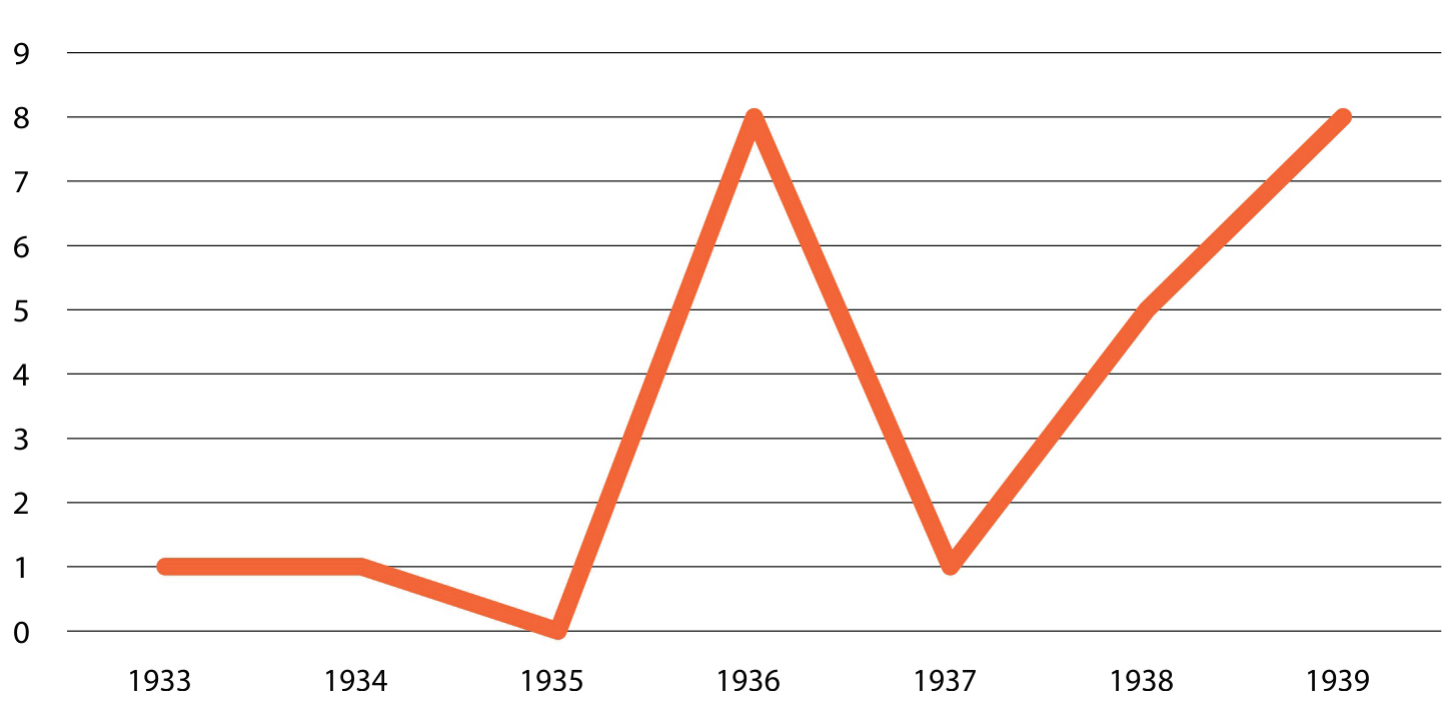
: Gráfico das menções ao termo “Josué de Castro” no jornal *A Noite*, 1930-1939 (Fonte: Hemeroteca da Biblioteca Nacional)

**Figura 2:**
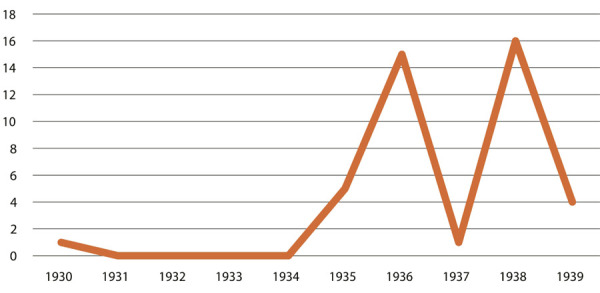
: Gráfico de menções ao termo “Josué de Castro” no jornal *Diário Carioca*, 1930-1939 (Fonte: Hemeroteca da Biblioteca nacional)

Predominantemente, os artigos de jornal da década de 1930 são anúncios de seus livros e comentários sobre o tema da alimentação – destaca-se o anúncio de uma conferência sobre a castanha do Pará (Castro, 14 ago. 1939). Castro é comumente citado e entrevistado em reportagens sobre essa temática, e ainda é possível encontrar nesse período os artigos que reproduzem seu célebre engajamento no debate sobre o salário-mínimo. É preciso lembrar também que Castro defendeu a criação de um serviço público dedicado à alimentação, em entrevista para o *Diário Carioca* (O problema..., 15 maio 1936). Castro se posiciona defendendo “a organização de um centro técnico, com gente entendida do assunto que formasse uma elite divulgadora das bases da alimentação” (p.3).

Nos anos subsequentes, Vargas instrumentalizaria essa ideia, tendo Castro à frente. Independentemente dessa temática, chama ainda a atenção o fato de Castro continuar a fazer crítica literária e publicar contos nos jornais cariocas, além de receber comentários de escritores como Orígenes Lessa (16 fev. 1936, p.4), que elogia seus livros: “Um especialista encontrará neles, ao lado da revelação de uma grande autoridade no assunto, um vulgarizador poderoso e seguro”. A publicação de contos reforça sua tendência de se engajar nas discussões nacionais, ou seja, a necessidade de sensibilizar a sociedade para a fome e para as desigualdades sociais. Nesse sentido, a redação de um livro didático de uma disciplina obrigatória como a geografia é mais um passo na direção de pautar o debate público.

Nesse contexto, é no campo literário que Castro encontra uma solução para sua vida editorial. Tanto Silva (2012), quanto Carvalho (2001) reproduzem cartas trocadas entre Josué de Castro e o escritor gaúcho Érico Veríssimo. As missivas de Veríssimo são afetuosas, indicando uma relação de amizade, provavelmente, nascida no Rio de Janeiro. Veríssimo agradece os elogios que Castro faz à sua obra *Olhai os lírios do campo*, de 1938, e escreve sobre as tratativas de uma possível tradução de *An outline of geography*, de Preston E. James (Carvalho, 2001). Veríssimo pergunta quanto Castro quer receber por página traduzida e quanto se deveria pagar a James pelos direitos da obra. A relação dos dois, provavelmente, é o nexo que explica a relação entre Castro e Porto Alegre: seus livros *Terapêutica dietética da diabete* (1936), *Festa das letras* (1937, em coautoria com Cecília Meireles), *Alimentação brasileira à luz da geografia humana* (1937) – prefaciado por Afrânio Peixoto, reitor da Universidade do Distrito Federal – e *Geografia humana: estudo da paisagem cultural do mundo* (1939) foram publicados pela Globo. Veríssimo foi secretário de redação da *Revista do Globo*, e, após a publicação de *Olhai os lírios...*, seu primeiro sucesso nacional*,* o escritor de Cruz Alta passaria ao posto de conselheiro, em um momento estratégico, quando a editora consolida sua abrangência nacional.

Paralelamente, observa-se no quadro de provas parciais da Universidade do Distrito Federal, publicado no *Jornal do Brasil* (Universidade..., 31 dez. 1936), que o curso de antropologia física era lecionado, em 1936, por Castro para o primeiro ano dos cursos de geografia, história, sociologia e ciências sociais. No final de 1936, com a saída de Pierre Deffontaines da universidade, o curso de geografia humana passou a ser lecionado por José Junqueira Schmidt, um climatólogo, e por Ernesto Street, figura ligada à federação das indústrias (Silva, 2012, p.571). Como colegas de destaque, encontramos no campo da geografia Fernando Antônio Raja Gabaglia, professor de fisiografia; Pierre Deffontaines, já mencionado; e Delgado de Carvalho, professor de história contemporânea. Dessa feita, vemos um Castro reconhecido como especialista em nutrição e como colega de várias figuras de peso para o processo de consolidação da geografia moderna, sendo que, após a saída de Deffontaines, a cadeira de geografia humana foi ocupada por professores que não eram da área. De acordo com Ferretti (2022), nesse período, Castro mantém correspondência com Max Sorre e Pierre Deffontaines, que reconhecem a pertinência do tema da geografia da alimentação, trocando informações e indicações de leitura sobre esse tema, bem como sobre o Nordeste e a geografia do Brasil.

É nesse contexto que Castro lança um livro didático que carrega no título a expressão *Geografia humana*, em uma editora de Porto Alegre, intermediado por Érico Veríssimo, e, o que é inusual, em vez de uma coleção que abrange todo ensino secundário, é publicado apenas um volume dedicado ao terceiro ano. Castro sabe da importância dos prefácios na legitimação simbólica de sua produção, e, portanto, não é fortuito que Preston E. James tenha assinado o preâmbulo de seu manual. Suas conexões francesas estavam bem consolidadas, seria interessante adquirir também contatos nos EUA, considerando que James aspirava ser um especialista sobre Brasil, assim como os colegas franceses, e havia, no âmbito da Associação dos Geógrafos Brasileiros, dado palestras sobre a colonização do sul do Brasil (Silva, 2012, p.567). Talvez essa tenha sido a ocasião em que eles se conheceram.

Parece crível que Castro aspirasse a ocupar a cadeira de geografia humana após a saída de Deffontaines, pois isso lhe conferiria maior visibilidade, além de um arcabouço teórico original para compreensão da fome, a partir da ótica da relação entre sociedade e natureza. Sabe-se que após a Intentona Comunista se deflagrou um processo de desmantelamento da Universidade do Distrito Federal (UDF), relacionando-a, bem como o seu idealizador Anísio Teixeira, à ameaça comunista. Apesar de não ser comunista, mas independente e crítico, o jornal *A Manhã*, do Rio de Janeiro, na primeira página da edição de primeiro de maio de 1935, publica uma entrevista com Castro cujo título é “Mais da metade dos trabalhadores brasileiros morre até os 30 anos por falta de alimentação”. O jornal era ligado à Aliança Nacional Libertadora, e seu diretor foi o comunista alagoano Pedro Mota Lima.

Castro ficou pouco tempo na cadeira de antropologia física da UDF, extinta em 1938, ocasião em que ele assume a disciplina de geografia física como professor adjunto. Desgostoso com a situação, Castro escreve ao reitor e ao ministro da educação Gustavo Capanema, em 1939, dizendo que foi à Itália estudar antropologia para se qualificar, aspirando se manter no cargo na recém-criada Universidade do Brasil; porém, a vaga dessa área acaba por ser ocupada por seu amigo Arthur Ramos. Dessa feita:

Josué de Castro só foi designado em 1940 para lecionar geografia humana como catedrático interino para substituir Pierre Deffontaines, que voltara à França. Como médico de Alzira Vargas, filha mais próxima a Getúlio Vargas, sua nomeação ocorreu, segundo Orlando Valverde, geógrafo e aluno de Castro, por indicação do próprio presidente durante o Estado Novo. ‘Eu vou lhe dizer com franqueza, eu achava ele brilhante, mas ele estava muito fraco em geografia propriamente. Eu sabia que ele era um médico em alimentação, mas em outras coisas ele estava apenas começando, como outros que foram nomeados por Getúlio’ (Leme, 2023, p.128-129; destaques no original).

Mais uma vez o capital social contou para que Castro encontrasse seu caminho. Assim, Castro fez uma espécie de jogo duplo, pois, dotado de uma agenda de pesquisa e um modo de conceber problemas interdisciplinares, ele almejava permanecer na universidade, fosse na geografia ou na antropologia, mesmo lecionando em cursos de extensão da área da medicina e ocupando cargos técnicos no governo, como no Departamento Nacional de Saúde Pública e na Comissão de Inquérito para o Estudo da Alimentação do Povo Brasileiro. De certo, Castro tinha competência para ser absorvido pelas áreas da saúde, como de fato o foi, ao fundar o Instituto de Nutrição da Universidade do Brasil. Entretanto, a inserção naquilo que atualmente chamamos de ciências humanas ofertava o instrumental metodológico e a legitimação científica para a afirmação de sua agenda de pesquisa, que claramente estava acoplada com o tema do combate à fome em uma perspectiva crítica e global.

Em paralelo, as cartas transcritas por Carvalho (2001) demonstram que, nesse período, Castro tinha uma relação de trocas profissionais e laços de amizade com Pierre Deffontaines e Francis Ruellan, este um geógrafo especialista em geomorfologia que permaneceu muitos anos no Brasil e teve um papel bastante ativo no Instituto Brasileiro de Geografia e Estatística (IBGE). Outro documento relevante apresentado por Carvalho (2001, p.58) é a carta de abril de 1938 enviada por Christovam Leite de Castro, então secretário do Conselho Nacional de Geografia (CNG), que comunica a Castro que foi permitido “o estágio em seus serviços a universitários discentes de cadeira ou cargo diretamente relacionado com a geografia”.

A missiva ainda denota que Christovam tratou do assunto diretamente com o reitor da UDF e que Leite de Castro iria receber Preston James em São Paulo, em um contato inicial que teria sido feito por Josué de Castro. Curiosamente, a contratação de Castro como professor adjunto na Faculdade de Filosofia do Distrito Federal, em 1940, é noticiada na *Revista Brasileira de Geografia* (Noticiário, 1940, p.666), o que pode ser, ao mesmo tempo, reflexo de suas boas relações com os colegas do CNG e sinal de seu reconhecimento intelectual. Não nos parece pouca coisa o CNG, que na época era vinculado ao Gabinete da Presidência da República, mobilizar-se para permitir que Castro lecionasse ou cooperasse cientificamente na geografia. Carvalho (2001, p.67-68) demonstra ainda que, em 1948, quando Castro já era professor de geografia concursado na Universidade do Rio de Janeiro, ele continuava a ter relações de amizade com Preston E. James, além de ser indicado, em 1949, para uma seleta delegação que objetivava representar o Brasil na primeira Reunião Pan-americana de Consulta sobre Geografia, que ocorreu em 12 de setembro de 1949, no Rio de Janeiro. Em paralelo, Ferretti (2022) demonstrou que na década de 1940 existiu uma profícua rede de cooperação entre Castro, Arthur Ramos e Roger Bastide, que reforçou e auxiliou este último antropólogo em suas pesquisas sobre a cultura afro-brasileira.

Nesse movimento, devemos lembrar o que Bourdieu (2011) pontua como expectativa de esperança: mesmo dedicado à nutrição, por ser um intelectual que atuava no campo literário, havia participado do curso pioneiro de geografia no Recife, interessava-se por temas afins da geografia, conhecia seus autores canônicos, tinha uma sólida formação científica que poderia ser reconhecida dentro e fora do campo da geografia, Castro poderia esperar que fosse um ocupante em potencial da cadeira de geografia humana após a saída de Deffontaines.

Em 1939, ele vai para a Itália, na Universidade de Roma, e visita ainda o Museu do Homem em Paris com o objetivo de aprimorar tanto sua formação na área de nutrição quanto na de antropologia – ocasião em que publica o livro *Alimentazione e acclimatazione umana nei tropici*. Pouco antes da partida, escreve para Gustavo Capanema, manifestando sua expectativa de ocupar a cadeira de antropologia na recém-criada Universidade do Brasil, mesmo que fosse professor adjunto de geografia humana (Leme, 2023, p.128; Barros, 2012, p.250). Como vimos, Castro galgaria um posto definitivo apenas em 1948, e, mais uma vez, tais atitudes poderiam ser vistas como esse jogo duplo de tentar ocupar um cargo universitário de prestígio, ora na antropologia, ora na geografia, para promover sua agenda política e científica de combate à fome, à pobreza e ao racismo. Nesse quadro de busca pelo reconhecimento, talvez a publicação do livro didático *Geografia humana* tenha sido um dos diversos lances que garantiram sua posição no campo da geografia.

## Geografia humana para crianças

O ensino básico sofreu modificações após a Revolução de 1930, pois a Reforma Francisco Campos, ocorrida em 1931, estabelece um processo de centralização de sua organização, com a fixação de regras gerais de funcionamento, seriação do currículo, normas de avaliação e frequência obrigatória. Assim:

Essas medidas procuravam produzir estudantes secundaristas autorregulados e produtivos, em sintonia com a sociedade disciplinar e capitalista que se consolidava, no Brasil, nos anos de 1930. A Reforma Francisco Campos, desta forma, marca uma inflexão significativa na história do ensino secundário brasileiro, pois ela rompe com estruturas seculares nesse nível de escolarização (Dallabrida, 2009, p.186).

Campos era influenciado pelos métodos ativos e individualizantes de ensino da Escola Nova, assim como expressiva parte dos geógrafos do Rio de Janeiro como Delgado de Carvalho e Everardo Backheuser, que se contrapunham aos antigos métodos mnemônicos e descritivos. Por força da lei (decreto 19.890, de 18 de abril de 1931), a reforma impôs o currículo do ensino de geografia do Colégio Pedro II para todo o Brasil. A grade de assuntos era bastante rígida, dando pouca margem para inovações nos livros didáticos, e, nesse sentido, o terceiro ano do curso de secundário da geografia deveria abordar geografia política e econômica do mundo e do Brasil. De acordo com Barros (2012), a disputa sobre educação nesse período polarizava, de um lado, os intelectuais progressistas inspirados pela nova escola e, de outro, figuras conservadoras defendendo que a Igreja e o ensino religioso conservassem um papel na educação. Nesse contexto, Castro ocupava uma posição interessante, pois era uma figura progressista, mas que tinha relações importantes com a Igreja. Durante o terceiro Congresso Eucarístico Nacional de 1939, ele perfila na comissão de honra (Culto..., 22 ago. 1939).

Após a reforma de Campos, o curso secundário tinha duração de sete anos e era dividido em dois ciclos. O ensino, no período, era elitista, não sendo acessível de uma maneira geral e irrestrita para a população. Castro redige manual para o terceiro ano, uma escolha que abrange temas familiares, como a questão racial, problemas de geografia econômica e produção de alimentos. Nos jornais, encontramos artigos e anúncios de palestras afins dessas temáticas – No *Diário Carioca*, do Rio de Janeiro, “Alimentação e aclimatação”, em 19 de julho de 1936, e “Aspectos biológicos da imigração e da colonização do Brasil”, em 15 de julho de 1938. O jornal *A Noite*, em 6 de outubro de 1938, também carioca, anuncia com mais informações a conferência “Aspectos biológicos da imigração e da colonização do Brasil”, que foi seguida de debates com Oliveira Viana, Fróes da Fonseca, Castro Barreto, Arthur Ramos e Bastos d’Avila, sendo o evento patrocinado pelo Instituto de Estudos Brasileiros.

O *habitus* da época demandava que tais manuais tivessem um caráter um tanto descritivo e se valessem do expediente de fotos, dados e tabelas para ilustrar, por exemplo, a produção de trigo ou café. *Geografia humana*, que Castro dedica a seu pai, chama atenção pela erudição, pois o autor cita direta ou indiretamente vários pensadores canônicos do campo da geografia: Emanuel de Martonne, Pierre Deffontaines, Georges Hardy, Ellsworth Huntington, Paul Vidal de la Blache, Halford Mackinder, Jean Brunhes, Jules Sion e Friedrich Ratzel, o que evidencia o seu domínio do campo e o alto nível intelectual proposto para um manual didático. Nota-se ainda o diálogo tanto com autores franceses quanto estadunidenses. Curiosamente, são mencionados ainda autores da antropologia como Leo Frobenius, Alfred Kroeber, Germano Correa e Oswald Spengler, para citar alguns. Dos brasileiros são citados os historiadores frei Vicente do Salvador, visconde de Porto Seguro, Basílio de Magalhães e o economista Roberto Simonsen.

Na introdução, a rubrica de Preston James tem seu peso, uma vez que o então jovem geógrafo fazia várias incursões no Brasil e constituía seu próprio capital social – de fato, a presença de Clarence Jones e Preston E. James constituíram espaços de cooperação que florescem nas décadas subsequentes na geografia do IBGE e do Rio de Janeiro (Schmidt, 2000). Como o próprio James (1935) admite, a influência da geografia francesa em seu pensamento é substancial, ou seja, ele se pauta por uma geografia regional, especializando-se na América Latina e no Brasil. Além do prefácio, o livro de Castro é ilustrado com mapas que são extraídos ou adaptados das obras de James – a saber: produção de trigo, arroz, milho, café, açúcar, beterraba, gado vacum e lanígeros, todos em escala mundial. O leitor atento encontrará ainda um paralelo entre a definição das regiões naturais mundiais postuladas por James (1935) e as de Castro (1939), classificadas de uma maneira simplificada e mais afeita à realidade brasileira.

No prefácio, após usar a interessante metáfora na qual o ser humano é como uma formiga caminhando em um tapete multicolorido matizado pelas diversas paisagens culturais, defender o método francês e a utilidade da geografia, o estadunidense afirma: “[este livro] não constitui simples compilação de fatos inexpressivos, mas, antes, [é] parte essencial do cenário onde se exercitam os seus estudos acerca dos urgentes problemas humanos do Brasil atual – especialmente nos campos da alimentação e da nutrição” (James, 1939, p.9). Esse balizamento mostra a aceitação do projeto intelectual de Castro ao usar a geografia humana como ferramenta de análise da alimentação.

Do ponto de vista do método, o manual didático é interessante, notadamente, porque Castro faz muitas definições científicas. Sobre o conceito de “raça”, ele pontua:

Com essas definições, fica bem claro que o termo raça não se cinge, de nenhum modo, aos limites artificiais das fronteiras políticas, levantadas pelas circunstâncias históricas, e independe dos conceitos de língua, religião, tradições, leis etc. Há grupos raciais que se estendem uniformemente por vários países, e há também países que comportam em sua população várias raças diferentes (Castro, 1939, p.32).

Castro conhecia os debates da antropologia cultural estadunidense e cita ideias de Kroeber e Haddon, desacreditando as teorias raciais mais ortodoxas. Ele afirma que “só por ignorância ou má-fé ainda hoje há quem fale em raça pura, e baseado nessa hipótese construa hierarquias com raças superiores e inferiores” (Castro, 1939, p.33). Mesmo que o autor evidencie o debate acerca da monogênese ou poligênese do homem, na época ainda não resolvido, ele relativiza as classificações raciais, cuja utilidade seria descrever diferenças anatômicas. Segundo Silva e Nunes (2017, p.3681) “Castro considerava o debate de raça pura ultrapassado e etnocêntrico, ressaltando que outros autores, como Manoel Bonfim, já haviam recolocado tais questões no debate nacional”, inclusive observando que a fome não se limita aos meios tropicais.

Sobre as classificações raciais, Castro se baseia em Kroeber, detalhando quais os critérios físicos considerados. Já Lima (1935, p.147), autor gaúcho de livros didáticos, detém-se menos em explicar os critérios de classificação e deixa transparecer uma série de preconceitos, por exemplo, definindo que existe a primazia da raça branca no Brasil, demonstrada pelos dados demográficos do período. O mais chocante é a reprodução de uma citação de Oliveira Viana, da obra *Populações meridionais do Brasil*, afirmando que todas as três “raças” brasileiras têm as mesmas oportunidades sociais, o mesmo acesso à terra e que não haveria nenhum tipo de lei discriminatória que se baseasse na raça (Lima, 1935, p.149). Nada mais fabuloso, cruel e distante da verdadeira realidade da população negra do Brasil, mesmo que tais concepções tenham solapado o pessimismo “racial”, como demonstrou Schwarcz (1993). Claramente, tais concepções eufemizaram a questão da dominação social em nosso país, que tem um cunho francamente racial.

Castro (1939), pelo contrário, buscava valorizar a cultura negra no Brasil, fosse incluindo um mapa no seu livro demostrando a procedência dos principais migrantes africanos que vieram para a América do Sul, fosse comparando os mocambos com as habitações nordestinas, demonstrando a contribuição profunda dessas culturas para a sociedade brasileira em termos populacionais e civilizacionais. O livro é permeado pelo uso do método comparativo, caro a Carl Ritter e utilizado por Friedrich Ratzel e Paul Vidal de la Blache. Se na geografia se comparam paisagens e regiões para sua melhor compreensão, na antropologia o mesmo procedimento é feito no tocante aos traços culturais.

Castro, crítico ao racismo, considera a cultura como fator explicativo mais importante em detrimento da “raça”. Vê os ibéricos como sagazes por se misturarem aos nativos e por adotarem seus hábitos de maneira mais flexível se comparado a outros processos de colonização, uma prática muito empregada na fase inicial da expansão imperial portuguesa (Bethencourt, 2018).

Parte do fundamento que o auxilia a analisar a questão racial se pauta no corolário inaugurado por Ratzel e repetido por la Blache e seus discípulos: o ser humano é um migrante, sempre movendo-se, trocando técnicas, plantas e animais, ao mesmo tempo adaptando-se ao meio (Lira, 2021). Tais migrações e adaptações criam e reconfiguram a relação entre sociedade e natureza, cujo resultado é paisagem humana. Possibilista, para Castro, os fatores culturais “coordenam e neutralizam” a influência do meio e dos fatores geográficos. Dessa forma, “é evidente que a técnica cultural resulta tanto das possibilidades materiais que o meio fornece como das intercorrências históricas, as migrações, os contatos do grupo com outros de cultura mais ou menos diferente etc.” (Castro, 1939, p.25). Outrossim, nosso autor defende que “a ciência que estuda e analisa a formação da paisagem cultural é a ‘Geografia humana’” (p.19; destaques no original). O tema volta à baila no seu estudo publicado na Itália, pois, segundo comentário de Ferretti (2022, p.37):

Enquanto Castro nega qualquer ação direta do clima nas sociedades humanas, o que é o determinismo ambiental, ele admite algumas influências das ‘condições mesológicas’ gerais, que deveriam ser investigadas, levando em conta a complexidade e diversidade dos ambientes tropicais em suas condições como umidade, equilíbrio exossistêmico e estruturas sociais (destaques no original).^1^

De forma notável, o estudo da geografia humana é dividido em uma parte dinâmica, interessada nas influências exercidas na relação sociedade/natureza – que podemos relacionar com o impulso migrante de Ratzel e la Blache – e outra parte estática, dedicada à compreensão do meio transformado – pura expressão da paisagem cultural (Castro, 1939, p.20). Nesse sentido, o hábitat e a alimentação são fatos essenciais: “O fato alimentação, que constitui a base da economia dos povos, foi a causa das primeiras migrações, da dispersão e da formação de vários núcleos de povoamento da terra. Só as terras férteis, com chuvas regulares, permitiriam, na Antiguidade, a formação de grandes aglomerados humanos” (p.24).

Essa construção é uma interpretação original do encadeamento do esquema de análise lablachiano, em que região (conjunto natural, histórico e humano homogêneo), paisagem (concreção da diversidade regional), gênero de vida (produção e hábitos culturais) são acrescidos à alimentação e se articulam para compreender a relação entre sociedade e natureza. Da perspectiva migrante, Castro valoriza as trocas culturais e reafirma a impossibilidade de raças “puras” e de idiomas isolados, demonstrando um posicionamento contra um evolucionismo único e linear:

O que se pensava antigamente ser uma diferença de grau – cultura mais baixa ou mais alta – a ser atingida através dos tempos, é, na realidade, uma diferença de qualidade de forma. Nasceu daí uma outra concepção, chamada morfologia das culturas, que as estuda como formas autônomas, e não como simples retratos de uma mesma forma através dos tempos (Castro, 1939, p.55).

Mesmo em defesa do relativismo cultural, entretanto, Castro era um homem de seu tempo, e em algumas passagens transparece o senso comum da época, como na citação: “Os conquistadores europeus, com um patrimônio cultural mais elevado que os povos conquistados na América, na Ásia e na Oceania, impuseram o seu idioma, em substituição às línguas nativas de suas colônias” (Castro, 1939, p.47). Não obstante, o livro é claramente contrário a uma visão racial eugênica, além de valorizar a cultura africana e popular, como na passagem em que ele cita Câmara Cascudo – outro célebre correspondente e colaborador científico (Ferretti, 2022) – para lembrar que os sertanejos não falam errado, mas remetem ao português arcaico (Castro, 1939, p.52).

Chama a atenção o tema da colonização e da aclimatação, pois vimos que tais assuntos são objeto de palestras nesse período. A colonização é um conceito compreendido de maneira ampla, em função de migrações humanas, e não está vinculado a uma ação imperialista. Assim: “Devemos fazer um estudo aprofundado da adaptação do homem aos novos meios para onde se desloca, e das técnicas de que lança mão para seu aproveitamento – fatos cujo estudo se acha englobado dentro do significado da palavra – ‘colonização’” (Castro, 1939, p.64). Aqui, deparamo-nos com uma temática importante, tanto para a geografia quanto para a antropologia, e Castro tenta harmonizar os conceitos de ambos os campos. Se a colonização se refere à ocupação do solo, à “adaptação biológica do elemento humano ao meio natural para onde é transportado, dá-se o nome de aclimatação. E ao processo de reajustamento entre os costumes e hábitos de vida do grupo que chega e os do grupo autóctone, dá-se o nome de aculturação” (Castro, 1939, p.64). Nosso autor adota um ponto de vista comedido, em que se faz um balanço das ações diretas e indiretas do meio geográfico, pois cultura e meio se influenciam; porém, nenhum elemento é determinante. Castro relembra o ponto de vista do geógrafo estadunidense Ellsworth Huntington – um determinista climático – de que as grandes civilizações teriam surgido nas zonas de clima tropical, para a seguir vaticinar: “[A] alimentação e gênero de vida são as molas que movimentam o processo de diferenciação dos grupos regionais” (Castro, 1939, p.70-71).

O pernambucano classifica ainda os tipos de colonização, como o de enraizamento, o de enquadramento (o colono só passa e é simples capataz) e o de posição (ocupação por interesse estratégico), o que revela uma sensibilidade e um conhecimento profundo do mundo colonial. Bizzo (2009) destacou o sentido civilizacional do pensamento de Castro, que comumente critica a mentalidade da ganância colonial, que não desenvolve o país de forma integral. Além disso, é necessária uma melhora total das características nacionais, pois a fome é causa da baixa produtividade do trabalho no Brasil, bem como sua parca integração territorial. O que se desvela aí é um projeto de modernização nacional que leva em conta a busca pela dignidade humana e as aclimatações, ou seja, as adaptações dos diversos gêneros de vida dos povos tradicionais, que de certa forma se contrapunham à cultura colonial, ou seja, do latifúndio da exploração do açúcar, do cacau ou do café. Ao comentar seu período de estudo na Itália, Ferretti (2022, p.36-41) destaca que Castro retoma o tema da aclimatação, elogia a adaptação dos ibéricos aos trópicos e, em pleno fascismo, destaca a miscigenação de europeus e africanos no contexto histórico do Mediterrâneo, além de criticar ações truculentas do imperialismo como massacres e a inserção de costumes “anti-higiênicos”.

Outro elemento importante é a referência aos fatos geográficos de economia destrutiva – exploração de recursos naturais –, de ocupação improdutiva – residências, por exemplo – e de economia produtiva – agricultura, pecuária etc.; uma classificação dos tipos de ocupação humana que remete à obra *Géographie humaine* de Jean Brunhes (1956), outro autor caro a James. Entretanto, é importante lembrar que, em 1929, existiu um programa de geografia no Colégio Pedro II profundamente inspirado por Brunhes (Barbosa, 2015). Isso pode ser a reverberação de um autor prestigiado no ensino de geografia e, igualmente, uma complexificação da discussão sobre a paisagem, usando-a como concreção didática. Ao definir os fatos essenciais da geografia humana, além de enfatizar a alimentação, Castro defende o estudo ecológico do hábitat, relacionando a construção das casas com os materiais disponíveis no meio, uma temática que se relaciona com a adaptação, a aculturação e o gênero de vida:

Quanto mais primitivo o tipo ‘de Habitação’, mais intensamente traduz este compromisso da arquitetura com o ‘quadro’ ecológico regional – donde a Habitação modesta, de caráter popular, possui muito maior significação para o geógrafo, que busca fixar os traços representativos da paisagem, do que os palácios e construções suntuosas (Castro, 1939, p.84-85; destaques no original).

Um raciocínio que podemos comparar com o de Tricart (1963), que segue o mesmo caminho e identifica o uso de materiais de construção industrializados como um indicador de modernização de áreas rurais e sua integração na economia. Sobre a habitação brasileira, ele compara:

Dessas influências, a mais nítida, sem nenhuma dúvida é a da cultura negra, importada da África com a escravidão. Basta olhar para o tipo clássico do mocambo (Fig. 50) e para a cabana típica do Congo, da Angola e da área do Golfo da Guiné, na África (Fig. 51), zonas essas que forneceram maior contingente de escravos ao Brasil, para que saltem aos olhos as analogias e homologias existentes entre esses dois padrões de habitação (Castro, 1939, p.93).

Nessa época, reconhecer as raízes africanas do Brasil era algo relevante, sendo que Castro tem um ponto de vista claramente cosmopolita que instiga os alunos a observar e pensar o mundo não europeu por meio de mapas e imagens. Certamente, seu treinamento na antropologia colabora para isso, o que o pode diferenciar dos outros geógrafos do período.

Sobre o hábitat, Castro aborda ainda as cidades, ressalta as diversas funções urbanas (turismo, cidade-porto, mineração etc.) e destaca a indústria como elemento propulsor da urbanização. Como seus contemporâneos, compara a cidade a um organismo vivo, aborda os círculos de expansão do perímetro urbano, refere-se às cidades americanas de crescimento rápido e ao advento das cidades planejadas, tomando Belo Horizonte como exemplo (Castro, 1939, p.102-104).

No capítulo sobre a geografia da circulação, sua estratégia didática fica clara: Castro parte da explicação geral para o regional, privilegiando exemplos brasileiros. Sobre os transportes, Castro se indaga a respeito das motivações dos deslocamentos, o que se produz e o que é transportado. Logo após, reconhece a importância dos tropeiros para a evolução econômica do país e enfatiza a evolução dos meios de transporte, notadamente a ferrovia e o automóvel. Cita Vidal de la Blache, que afirma que os trilhos vivificam tudo o que tocam, mas as estradas de ferro tendem a homogeneizar as paisagens, pois usam as mesmas estruturas, geralmente acompanhadas de linhas de telégrafo, armazéns etc., um fenômeno que abrange vários rincões do mundo. Tais avanços mudam a geografia da alimentação: “Com o advento das estradas de ferro aperfeiçoadas, todas as cidades se suprem desses elementos básicos da alimentação, em zonas rurais mais ou menos distantes” (Castro, 1939, p.127). A drenagem mais intensa dos excessos produtivos reforça o sistema agroexportador e estimula o surgimento de cidades encruzilhadas.

Na parte final do manual sobre a produção agrícola, Castro dá vazão à sua concepção de geografia da alimentação. Para nosso autor, a agricultura é vista como um advento civilizacional e a alimentação é compreendida como inerente ao complexo cultural dos povos. Ademais, “o estudo da distribuição geográfica das culturas alimentares constitui um capítulo importantíssimo em antropogeografia, uma vez que a geografia botânica representa o elo intermediário entre a geografia física e a econômica” (Castro, 1939, p.141). Complementa ainda: “Cada povo, com seus hábitos alimentares próprios, organiza sua conduta econômica de um modo particular, criando uma técnica peculiar de cultivo, de colheita, de preparo e de utilização dos alimentos, inerente ao seu complexo cultural” (p.143). Cabe lembrar que o conceito de complexo cultural é bastante caro e conhece várias definições no campo da antropologia.

Assim, sua síntese apresenta aos alunos a relação entre clima e alimentação (distribuição de plantas e animais), além dos tipos regionais de alimentação ou das culturas alimentares, ressaltando a diferença paisagística da monocultura e da policultura. A monocultura representa um empobrecimento da alimentação regional, apesar da maior eficácia produtiva. Ao citar o médico argentino Pedro Escudero, segundo Davies (2023) um autor que o influenciou, os grãos seriam importantíssimos por seu valor nutricional e pela facilidade de transporte. O autor aborda a cultura de trigo, arroz, milho, café, cacau, chá, açúcar e vinho, narra sua história, destaca as principais áreas produtoras, seu consumo e sua paisagem. Para o trigo e o milho, Castro se debruça sobre suas características nutricionais didaticamente, sendo que tais explicações têm uma estratégia expositiva semelhante à de *Geografia da fome*.

Sempre munido de mapas e dados estatísticos, Castro aborda ainda a criação de animais e as plantas de valor comercial (algodão, borracha, madeira, tabaco, gado, lanígeros e seda), para a seguir finalizar o livro didático falando de maneira sucinta sobre a produção mineral, abordando o carvão, o petróleo, o ferro e outros minerais com uma lista dos principais países produtores. Além disso, escreve sucintamente sobre a geração de energia.

Cabe um último paralelo com seu colaborador Preston E. James, que, junto com a professora Gertrude Whipple, da Universidade Wayne, publica uma série de manuais de geografia na década de 1950. Mesmo Castro recebendo o prêmio F.D. Roosevelt dos EUA, em 1952, e a medalha da academia de ciências da URSS, em 1963, durante a Guerra Fria seu compromisso foi sempre com os povos do chamado Terceiro Mundo. O livro de James e Whipple (1951, p.VI), entretanto, advoga um claro engajamento do ensino da geografia:

O professor de geografia está bem equipado para comparar o comunismo soviético e nossa democracia como modo de vida e assim demonstrar a superioridade da democracia em suprir ambas necessidades da humanidade, a moral e a material. É o geógrafo, mesmo mais do que o economista, que está equipado para expor as falácias econômicas do comunismo. Mas, para fazê-lo, ele deve estar livre para contar a verdade sobre a União Soviética.

Tal comparação ilustra as dimensões e nuances que o ensino de geografia pode adquirir. Castro, infelizmente, após a publicação de *Geografia humana*, não se dedicou mais aos manuais da educação básica.

## Considerações finais

Nossa exposição permite problematizar dois comentários feitos por geógrafos, em entrevistas organizadas por Silva (2012): (1) o de Orlando Valverde, de que Castro, nos anos 1930, sabia pouca geografia; a análise do livro didático prova o contrário; e (2) a afirmação de Aziz Ab’Sáber de que *Geografia humana* não seria um livro original, apesar do alto padrão se comparado aos seus contemporâneos. Certamente, as rupturas científicas não decorrem de livros didáticos, mas é possível reconhecer que o manual é original ao apresentar conceitos e ideias complexas da geografia, problematizar o racismo, o determinismo climático e constituir um diálogo com a antropologia.

Castro traduz para o ensino de geografia a nova perspectiva, pautada na ecologia humana e nos aportes da geografia francesa: a raça e o clima são elementos secundários da explicação da geografia humana; a chave explicativa é o possibilismo, as relações entre sociedade e natureza em um mundo que se moderniza e se integra. Assim, Castro alia as reflexões oriundas da nutrição e da fisiologia aos métodos da moderna geografia francesa. É notório que a geração de Pierre George, oriunda dessa escola, modernizou o pensamento de Vidal de la Blache, tendo em conta o marxismo, e criou uma geografia ativa, ou seja, aplicada para a solução dos problemas socias. Castro fez o mesmo, ainda na década de 1940, pois compreendia que o método geográfico poderia revelar o problema da fome em sua totalidade (Castro, 1948, p.457) e, diferentemente de Pierre George e Yves Lacoste, defendeu uma postura essencialmente antimalthusiana (Ferretti, 2019). Dessa feita, sua proposta de ensino valoriza a cultura e os hábitos nacionais e populares, permitindo uma contribuição crítica na disputa pelos imaginários geográficos sobre a imagem do Brasil e do mundo. Nesse sentido, a literatura, a história e a geografia sempre tiveram um papel em consolidar o imaginário sobre a comunidade e o território (Anderson, 2008), o que nos permite vislumbrar a posição de Castro no campo da geografia ao defender uma agenda de forte conteúdo nacional e popular. Detrás de sua proposta de ensino está sua proposta civilizacional para o Brasil (Bizzo, 2009).

Além do manual didático proporcionar um ensino empático e crítico para os padrões da época, sua análise nos dá o justo retrato de uma síntese da atuação de Castro no campo da geografia e da antropologia, além de ser um preâmbulo para várias ideias e estratégias de argumentação que seriam utilizadas em *Geografia da fome.* Ou seja, *Geografia humana* é um processo de experimentação e maturação intelectual que reafirmou seu projeto intelectual de uma geografia da alimentação, que foi encaminhada para o tema da fome.

Castro, na década de 1930, era um polímata preocupando-se com uma inserção internacional, quase sempre interessado em estudar as especificidades da realidade brasileira ou dos países da periferia. À luz da teoria dos campos, cabe ainda um comentário sobre o que chamamos de jogo duplo, ou seja, sua inserção no campo da medicina e nutrição, paralelamente à sua atuação ambígua, ora na antropologia, ora na geografia, que se atenua com a publicação de *Geografia da fome*. Apesar de existir uma rápida internacionalização de sua obra (Davies, 2023), nota-se uma atuação fraca nas sociedades de geografia, e, a partir de meados da década de 1950, o seu engajamento no campo político como deputado pode tê-lo afastado de uma atuação efetiva no campo, o que tem por consequência sua falta de reconhecimento como cânone da geografia. Mesmo a geografia crítica, na década de 1970, não deu a devida atenção à sua contribuição, o que tem como resultado um reconhecimento recente, que concorre com o de sociólogos que se dedicaram ao tema da fome. Sua posição no campo da geografia é bastante atípica e, até certa medida, distinta daqueles geógrafos de sua geração que se profissionalizaram e se especializaram, obtendo, assim, um capital simbólico mais estável e duradouro, mesmo que mais restrito ao campo.

Cabe destacar, no entanto, que Castro teve uma influência fundamental para Milton Santos, que se considerava seu discípulo e que nos primeiros anos de seu exílio também se dedicou ao tema da alimentação. A epígrafe escolhida para esse texto pode ser lida como uma trágica premonição do destino de Josué de Castro, um intelectual com inserção internacional, mas que nunca abriu mão de sua agenda científica a favor de modismos e que foi intransigente no seu esforço de compreensão do Brasil e da periferia do mundo. Nesse sentido, seu manual didático é um passo na luta por um Brasil mais justo, sem fome e desenvolvido.

## NOTA

1 Nessa e nas demais citações em língua inglesa, a tradução é livre.

## References

[B1] (1934). ALIMENTAÇÃO e raça. A Noite.

[B2] AMORIM Helder Remigio de (2022). Josué de Castro: um pequeno pedaço incomensurável.

[B3] ANDERSON Benedict (2008). Comunidades imaginadas.

[B4] ANTUNES Charles da França (2023). A Associação dos Geógrafos Brasileiros - AGB: origens e transformações.

[B5] BARBOSA Danyele Vianna (2015). Os programas de ensino de geografia do Colégio Pedro II de 1926 a 1951: a transição do caráter corográfico ao discurso científico a serviço de um novo projeto de escola e de Brasil.

[B6] BARROS Luitgare Oliveira Cavalcanti, Silva Tânia E.M. da (2012). Josué de Castro.

[B7] BERDOULAY Vincent (2003). A abordagem contextual. Espaço e Cultura.

[B8] BETHENCOURT Francisco (2018). Racismos: das cruzadas ao século XX.

[B9] BIZZO Maria Letícia Galluzzi (2009). Ação política e pensamento social em Josué de Castro. Boletim Museu Paraense Emílio Goeldi.

[B10] BIZZO Maria Letícia Galluzzi, LIMA Nísia Trindade (2010). O projeto civilizatório nacional do Instituto de Nutrição da Universidade do Brasil (1946-1960). Perspectivas.

[B11] BOURDIEU Pierre (2011). A distinção: crítica social do julgamento.

[B12] BOURDIEU Pierre (2003). Os usos sociais da ciência.

[B13] BOURDIEU Pierre (2001). Science de la science et réflexivité.

[B14] BRUNHES Jean (1956). Géographie humaine.

[B15] CAMPELLO José (1936). Alimentação e raça. Diário Carioca.

[B16] CARNEIRO Humberto (1935). Alimentação e raça. Diário Carioca.

[B17] CARVALHO Antônio Alfredo Teles de (2008). O pão nosso de cada dia nos dai hoje... Josué de Castro e a inclusão da fome nos estudos geográficos.

[B18] CARVALHO Antônio Alfredo Teles de (2001). Josué de Castro na perspectiva da geografia brasileira - 1934-1956.

[B19] CASTRO Josué Apolônio de (1948). Áreas alimentares do Brasil. Boletim Geográfico.

[B20] CASTRO Josué Apolônio de (1939). Geografia humana: estudo cultural da paisagem.

[B21] CASTRO Josué Apolônio de (1939). A Noite.

[B22] CASTRO Josué Apolônio de (1935). Alimentação e raça.

[B23] CASTRO Josué Apolônio de (1933). O problema da alimentação no Brasil.

[B24] CULTO católico (1939). A Noite.

[B25] DALLABRIDA Norberto (2009). A reforma Francisco Campos e a modernização nacionalizada do ensino secundário. Educação.

[B26] DAVIES Archie (2023). A world without hunger: Josué de Castro and the history of geography.

[B27] (1939). ENTREVISTA com Josué de Castro. Vamos Ler!.

[B28] FERRETTI Federico (2022). Social tropicalism, engaged geographies and the Brazilian "hub". Revue d'Histoire des Sciences Humaines.

[B29] FERRETTI Federico (2019). A coffin for Malthusianism: Josué de Castro's subaltern geopolitics. Geopolitics.

[B30] JAMES Preston Everett, Castro Josué A. de (1939). Geografia humana: estudo cultural da paisagem.

[B31] JAMES Preston Everett (1935). An outline of geography.

[B32] JAMES Preston Everett, WHIPPLE Gertrude (1951). Our earth and man - Euroasia and the world.

[B33] LEME Adriana Salay (2023). Josué de Castro e a fome: gênese e gestão de uma questão social no Brasil.

[B34] LEME Adriana Salay (2021). Josué de Castro e as metamorfoses da fome no Brasil, 1932-1946. História, Ciências, Saúde - Manguinhos.

[B35] LESSA Orígenes (1936). Diário Carioca.

[B36] LIMA Afonso Guerreiro (1935). Geografia secundária - 3ª série.

[B37] LIRA Larissa Alves (2021). Pierre Monbeig e a formação da geografia no Brasil.

[B38] MACHADO Mônica Sampaio, ALVES Camila, AZEVEDO Gustavo (2010). Josué de Castro e o Brasil: primeiras considerações.

[B39] MELO Marcos Antônio (1991). Josué de Castro: geógrafo da fome.

[B40] MELO Normando Jorge de Albuquerque, Silva Tânia E.M. da (2012). Josué de Castro.

[B41] MENDES Thomaz de Figueiredo (1935). Diário Carioca.

[B42] MENDONÇA Marina Gusmão de (2021). O combatente da fome: Josué de Castro - 1930-1973.

[B43] NOTICIÁRIO (1940). Revista Brasileira de Geografia.

[B44] (1936). O PROBLEMA da alimentação no Brasil. Diário Carioca.

[B45] PINTO Edgard Roquette (1936). Diário Carioca.

[B46] SCHMIDT Roberto (2000). A geografia e os geógrafos do IBGE no período 1938-1998.

[B47] SCHWARCZ Lilia (1993). O espetáculo das raças.

[B48] SILVA Mercês de Fátima dos Santos, NUNES Everardo Duarte (2017). Josué de Castro e o pensamento social brasileiro. Construtores da Saúde Coletiva.

[B49] SILVA Tânia Elias Magno da (2012). Josué de Castro.

[B50] TRICART Jean (1963). Cours de géographie humaine: L'habitat rural.

[B51] UNIVERSIDADE do Distrito Federal (1936). Edital n. 49 ED. Jornal do Brasil.

[B52] VASCONCELOS Francisco de Assis Guedes de (2001). Fome, eugenia e constituição do campo da nutrição em Pernambuco: uma análise de Gilberto Freyre, Josué de Castro e Nelson Chaves. História, Ciências, Saúde - Manguinhos.

